# Predictive value of bone turnover markers and thyroid indicators for bone metabolism in GD patients after treatment

**DOI:** 10.3389/fendo.2024.1301213

**Published:** 2024-04-29

**Authors:** Mengxue Su, Jinyan Chai, Wei Zheng, Qiang Jia, Jian Tan, Yajing He, Ruiguo Zhang, Jianlong Men, Wei Liu, Tao Shi, Jing Ren, Liyan Dong, Luyi Liu, Zhaowei Meng

**Affiliations:** ^1^ Department of Nuclear Medicine, Tianjin Medical University General Hospital, Tianjin, China; ^2^ Precision Medicine Center, Tianjin Medical University General Hospital, Tianjin, China; ^3^ Department of General Surgery, Tianjin Medical University General Hospital, Tianjin, China; ^4^ George’s School, Middletown, RI, United States

**Keywords:** bone turnover markers (BTMs), hyperthyroid, osteocalcin, P1NP, procollagen type 1 N-terminal propeptide, CTX (C-terminal telopeptide of type 1 collagen)

## Abstract

**Purpose:**

To investigate the relationship between bone turnover markers (BTMs) and thyroid indicators in Graves’ disease (GD) and to further assess predictive value of changes in early stage retrospectively.

**Methods:**

We studied 435 patients with GD and 113 healthy physical examiners retrospectively and followed up these two groups of patients after 6 months. We investigated the correlations between BTMs and other 15 observed factors, and analyzed the predictive value of FT_3_ and FT_4_ before and after treatment (FT_3_-P/FT_3_-A, FT_4_-P/FT_4_-A) on whether BTMs recovered.

**Results:**

The levels of thyroid hormones and BTMs in GD group were significantly higher than those in control group (P < 0.05) and decreased after 6 months of treatment. FT3, W, Ca and ALP were independent factors in predicting the elevation of OST. Duration of disease, FT3, TSH and ALP were independent factors in predicting the elevation of P1NP. Age, duration of disease, TRAb and ALP were independent factors in predicting the elevation of CTX-1. The AUC of FT_3_-P/FT_3_-A and FT_4_-P/FT_4_-A for predicting OST recovery were 0.748 and 0.705 (P < 0.05), respectively, and the cut-off values were 0.51 and 0.595. There was no predictive value for P1NP and CTX-1 recovery (P > 0.05).

**Conclusion:**

BTMs were abnormally elevated in GD and were significantly correlated with serum levels of FT3, FT4, TRAb, Ca, and ALP. FT_3_ decreased more than 51% and FT_4_ dropped more than 59.5% after 6 months of treatment were independent predictors for the recovery of BTMs in GD.

## 1 Introduction

Graves’ disease(GD) is an autoimmune form of hyperthyroidism with a global prevalence of 2-4 per 10,000 (1). In a genetic and environmental background, GD is characterized by symptoms of hypermetabolism and over-secretion of thyroxine. It is also associated with other kinds of autoimmune diseases and more common in women (with a male to female ratio of 1:10) (1–4). Thyroid hormone plays a crucial role in the regulation of chondrocytes, osteoblasts and osteoclasts, and is essential for skeletal linear growth and bone mass maintenance (5). However, in the state of GD, over-secretion of thyroid hormones often leads to high-turnover osteoporosis as it accelerates bone resorption and bone formation (6). Even patients with subclinical hyperthyroidism are at risk of this (7). Besides, bone metabolism is influenced by many factors, including gender, age, diet, sex hormones, parathyroid hormone (PTH), vitamin D and calcium and phosphorus homeostasis (8–12). Some of these factors may be impacted by thyrotoxicosis, leaving the exact mechanism unknown.

The most common measurement currently used to diagnose osteoporosis is bone mineral density (BMD) (10). This screening is only able to detect the quantity of bone tissue rather than being manifestly sufficient to measure the quality and vitality of the bone (13). In recent years, bone turnover markers (BTMs) have been used clinically as a complementary indicator to BMD due to higher sensitivity and specificity (14). BTMs are consisted of bone formation markers, including Osteocalcin (OST) and Procollagen I N-Terminal Propeptide (P1NP), and bone resorption markers, including C-terminal telopeptides of Type I collagen (CTX-1). In a study with 27 hyperthyroid patients, the level of serum OST was significantly higher in these hyperthyroid patients than in normal subjects than in normal subjects (15). In another study from Greece, Konstantinos et al. showed that both hyperthyroidism and hypothyroidism negatively affect bone mass (16). The studies of BTMs and GD mentioned above were all cross-sectional with limited number of cases and focused mainly on the relationship between the level of thyroid function and OST.

On the basis of the above researches, this study aimed to analyze the relationship between various indexes and bone metabolism indexes of GD patients. Whether the degree of early hypothyroidism due to ^131^I therapy can predict the recovery of bone metabolic markers, and provide a new research idea for the follow-up, guiding treatment and avoiding the development of osteoporosis.

## 2 Materials and methods

### 2.1 Study subjects

The present study was designed and completed at the Nuclear Medicine Outpatient and Inpatient Departments of Tianjin Medical University General Hospital. Patients were recruited between October 2020 and October 2022. Inclusion criteria: (1) Compliance with the diagnostic criteria for hyperthyroidism in the Chinese Guidelines (17) for the Diagnosis and Treatment of Thyroid Diseases, i.e.: goiter by ultrasound and palpation; signs and symptoms of hypermetabolism; decreased levels of thyroid stimulating hormone (TSH); positive autoimmune antibodies against TSH-receptors (TRAb); Increased thyroid uptake of iodine 131 (RAIU). (2) premenopausal women (aged 18 to 45) and men aged 18 to 50; (3)drug-naive cases of hyperthyroidism or relapse cases without ATD therapy in the last 1 months. Exclusion criteria: (1) Diagnosed osteoporosis or combination of other kinds of disorders affecting bone metabolism, such as diabetes, collagen metabolic disorders, or bone and joint disorders; (2) Combination of significant organ insufficiency, malignancy, infectious diseases, or psychiatric diseases; (3) Women with irregular menstruation, early menopause syndrome (MPS), or in the stage of pregnancy or lactation; (4) Corticosteroid therapy and vitamin D and/or calcium supplements within the last 1 years.

The study consisted of 2 visits. During the first visit (baseline), all patients from the inpatient unit underwent a thorough examination. Each completed a questionnaire covering gender, age, medical history, family history of fracture, comorbid medications, weight and height, and underwent a physical examination (the following morning, fasting status): blood samples were taken to measure free triiodothyronine (FT_3_), free thyroxine (FT_4_), TSH, TRAb, thyroglobulin antibody (TgAb), thyroid peroxidase antibody (TPOAb), 25-hydroxyvitamin D (25(OH) -VD), serum calcium (Ca), serum phosphorus (P), alkaline phosphatase (ALP), RAIU, BTMs (including: OST, P1NP and CTX-1) and thyroid ultrasonography. Patients from outpatient clinics had only blood samples taken to measure FT_3_, FT4, TSH, TRAb and BTMs (including: OST, P1NP and CTX-1) due to the limitations of the examination environment. All patients included in the study received treatment for hyperthyroidism after the first examination (baseline), including antithyroid drugs as well as ^131^I therapy. Treatment protocols were based on the guideline for the treatment of hyperthyroidism. At the 6th month after the start of treatment, we asked the patients to come to the hospital for follow-up examinations including: FT3, FT4, TSH, OST, P1NP, and CTX-1.

OST was considered abnormally elevated if it was greater than the 95th percentile (P95) of serum OST levels of control group. Same for P1NP and CTX-1. We defined the criteria for “OST recovery” as either a decrease in OST levels greater than 30% after treatment (2-fold laboratory error) or OST levels within the normal range at follow-up. Same for P1NP and CTX-1. Due to the limitations of the testing laboratory instrumentation, the test results of some indicators were shown as greater than the highest value. Therefore, in the statistics, we expressed such data as the highest value.

Meanwhile, matched by gender and age, we selected some healthy physical examiners from the same period as the control group, with the same inclusion criteria(2) as those for the hyperthyroidism group and exclusion criteria (1~4).

This study was approved by the Ethics Committee of the Tianjin Medical University General Hospital (IRB2023-KY-090).

### 2.2 Study conduct

**Figure 1:**
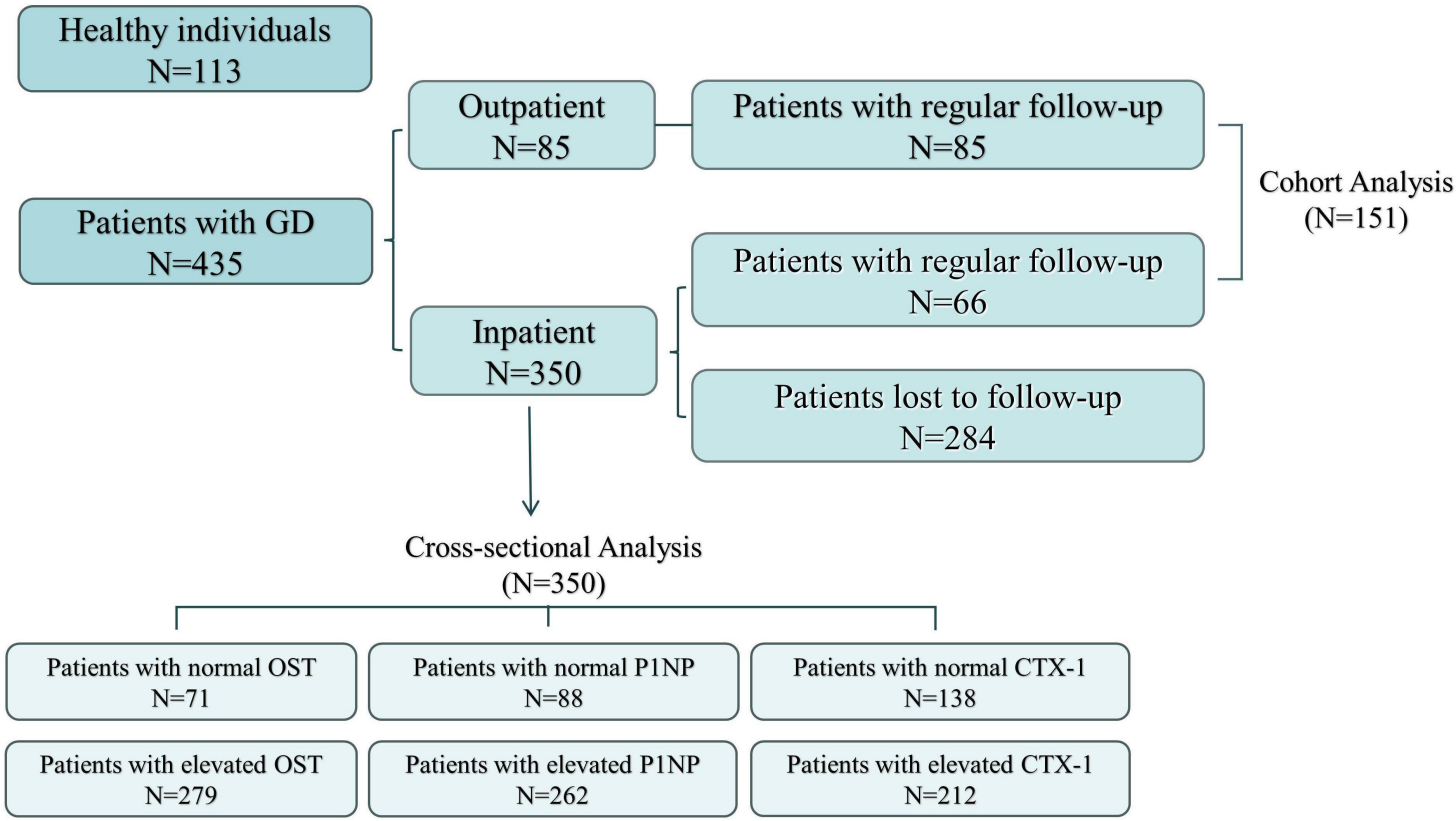
Flowchart of the participants.

There were 435 patients (350 patients from the nuclear medicine ward and 85 from the nuclear medicine outpatient clinic) who met the above criteria. Due to medicare financial burden of many cases, only 151 patients eventually completed the second measurement of the whole set of factors during the 2nd visit. In addition, there were 113 healthy individuals included in the control group. Further, written informed consent was obtained from all patients for participating the study. The selection and drop outs of study participants were shown in Flowchart Figure (**Figure 1**).

### 2.3 Serum hormone measurements and RAIU assays

Thyroid volume was assessed by ultrasound (GE Logiq 400 Pro, GE Healthcare). FT3, FT4 and TSH levels were determined by chemiluminescence immunoassay (Abbott Laboratories, Chicago, IL, USA). TgAb, TPOAb and TRAb levels were determined by electrochemiluminescence immunoassay (Roche Diagnostics GmbH, Mannheim, Germany). 25(OH)-VD levels were determined by chemiluminescence immunoassay (Aichitect i2000, Abbott, America). Serum calcium and alkaline phosphatase levels were determined by colorimetric assay (LABOSPECT 008 AS, Japan). BTMs levels were determined by electrochemiluminescence immunoassay (Roche Cobas e 601, Germany). Radioactivity was measured using a Na^131^I nuclear multifunctional instrument (MN-6300XT Apparatus, Technological University, China). Counting was conducted for 1 min with the single-channel analyzer in the differential mode, and 74 kBq of ^131^I was administered 24 h before the RAIU measurement. The thyroid radioactivity uptake was calculated using background radiation correction and a reference standard: thyroid uptake=(neck counts—the remainder of the body counts)/(reference counts—room counts). The RAIU of the thyroid was determined dynamically at 24, 48, and 72 h to determine the highest RAIU (RAIUmax) and Tef (18).

### 2.4 Statistical analysis

Results were presented as median (P25,P75) because of non-normal distribution of factors. The differences of BTMs between GD group and control group were determined by Rank Sum test. Spearman Correlation Analysis, Univariate Analysis and Multivariate Logistic Regression Analysis were used to identify the indicators that have significant impacts on BTMs. Alignment Diagram and Decision Curve Analysis(DCA) were used to analyze the clinical decision effectiveness of the index. The predictive value of each test index for abnormally elevated BTMs and the diagnostic value of the degree of decrease in thyroxine (FT_3_-P/FT_3_-A、FT_4_-P/FT_4_-A) for the recovery of BTMs were assessed by plotting Receiver Operating Characteristic (ROC) curves. Cut-off values were selected based on the Youden index. We considered the difference to be statistically significant when P < 0.05. Statistical analysis was performed by Origin 2022 and SPSS 26.0 (IBM Corp, Armonk, NY, USA).

## 3 Results

### 3.1 BTMs (OST, P1NP and CTX-1) of GD group and control group

**Figure 2:**
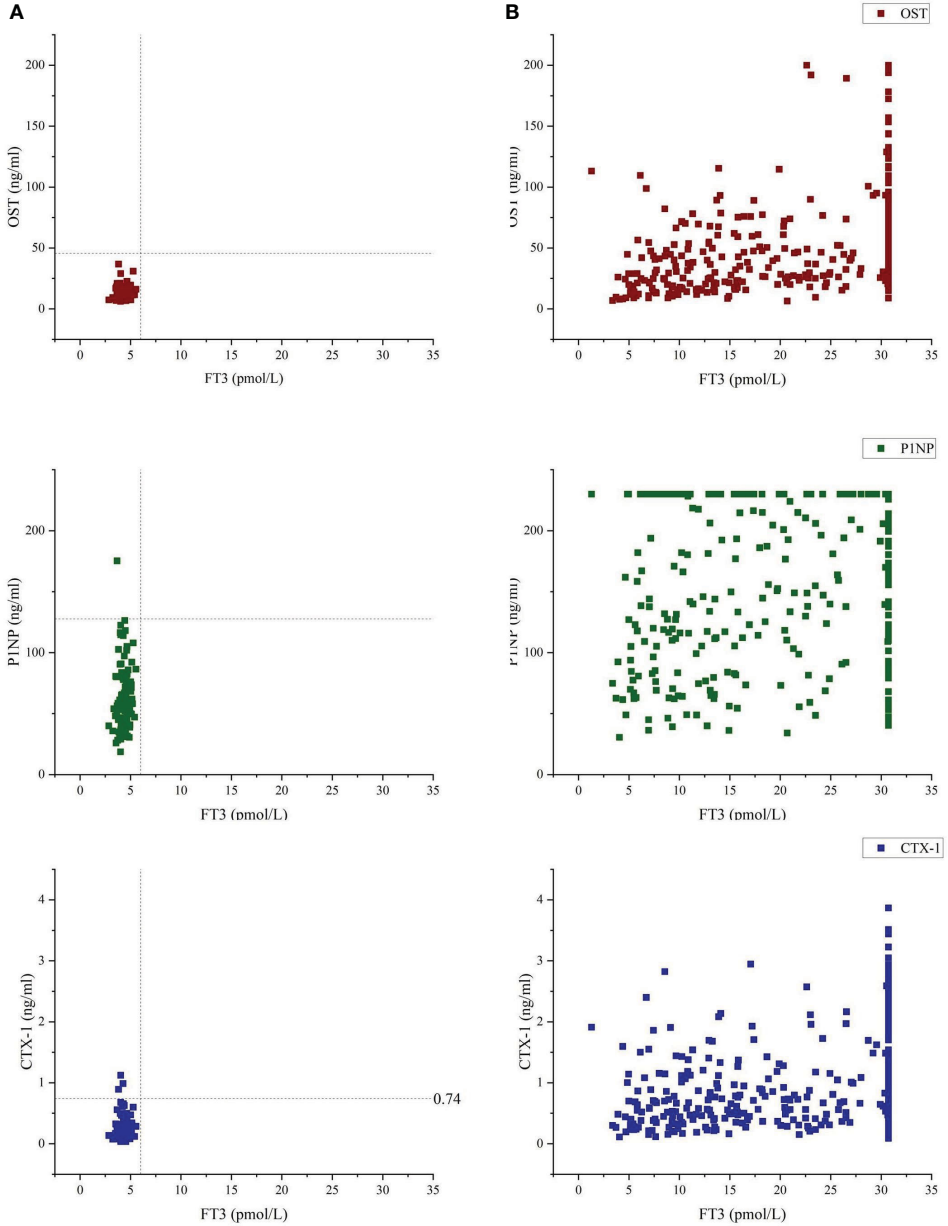
Bone turnover markers (OST, P1NP and CTX-1) vs free-T3 of Control group **(A)** and GD group **(B)**. Vertical dashed line indicates 6.01 pmol/l, which was the upper reference limit for free-T3, using the laboratory method of our hospital's clinicallaboratory. Median and range for OST were 11.00(8.85-13.7) and 41.45(23.38-71.70) of Control group and GD group, respectively. Median and range for P1NP were 57.40(44.45-75.25) and 191.45(114.43-230.00) of Control group and GD group, respectively. Median and range for CTX-1 were 0.197(0.134-0.306) and 0.771(0.471-1.352) of Control group and GD group, respectively.

The differences in gender and age between the GD group and the control group were not statistically significant (P > 0.05). Compared with the control group, the serum FT3 and FT4 levels were significantly higher (P < 0.05) and TSH levels were significantly lower (P < 0.05) in GD group. Moreover, the levels of OST, PINP and CTX-1 in the GD group were obviously higher than those in the control group (P < 0.05) (**Table 1**, **Figure 2**).

**Table 1:** Comparison of Patient characteristics, Thyroid hormone and BTMs between Control group and GD group.

	Control group (n=113)	GD group (n=350)	Z	P-Value
Gender (Male/Female)	34/79	103/247	-0.133	0.894
Age	33 (28,38)	33 (28,38)	-0.262	0.793
FT3 (pmol/L)	4.33 (4.05,4.65)	22.84 (11.90,30.72)	-15.648	**<0.001**
FT4 (pmol/L)	12.67 (11.94,13.80)	32.31 (22.44,42.82)	-14.270	**<0.001**
TSH (uIU/mL)	1.783 (1.180,2.560)	0.004 (0.004,0.004)	-18.395	**<0.001**
OST (ng/ml)	11.00 (8.85,13.7)	41.45 (23.38,71.70)	-13.959	**<0.001**
P1NP (ng/ml)	57.40 (44.45,75.25)	191.45 (114.43,230.00)	-13.540	**<0.001**
CTX-1 (ng/ml)	0.197 (0.134,0.306)	0.771 (0.471,1.352)	-13.090	**<0.001**

Bold values in the table indicate that these indicators are statistically significant.

### 3.2 Correlations of BTMs and various indicators

The correlations of BTMs with indices of thyroid function, thyroid weight (W) and some serological indicators were shown in **Table 2**. It could be observed that there was statistically significant correlations between most of the indicators included in the statistics and the BTMs. Specifically, OST, as well as CTX-1, was negatively correlated to age, duration of disease and TSH levels and positively correlated to serum FT3, FT4, TRAb, 25(OH)-VD, Ca and ALP levels and W. Besides, OST was positively correlated to RAIU. P1NP was negatively correlated to duration of disease and TSH levels and positively correlated to serum levels of FT3, FT4, TRAb, 25(OH)-VD, Ca and ALP levels, W and RAIU. Meanwhile, both P1NP and CTX-1 were more likely to be elevated in men. OST, CTX-1, as well as P1NP were closely related to duration of disease, FT3, FT4, TSH, TRAb, ALP levels (r>-0.2).

**Table 2:** Correlations of BTMs and various indicators.

	Gender	Age	Duration of disease	FT3	FT4	TSH	TRAb	TgAb	TPOAb	RAIU	Thyroid weight	25(OH)-VD	Ca	ALP
OST														
r	-.067	-.143^**^	-.293^**^	.476^**^	.483^**^	-.254^**^	.499^**^	.017	.000	.113^*^	.187^**^	.151^**^	.322^**^	.684^**^
p	.214	**.008**	**.000**	**.000**	**.000**	**.000**	**.000**	.753	.998	**.034**	**.000**	**.005**	**.000**	**.000**
P1NP														
r	-.115^*^	-.068	-.326^**^	.390^**^	.365^**^	-.281^**^	.419^**^	-.013	-.036	.106^*^	.127^*^	.162^**^	.296^**^	.743^**^
p	**.032**	.207	**.000**	**.000**	**.000**	**.000**	**.000**	.808	.505	**.047**	**.017**	**.002**	**.000**	**.000**
CTX-1														
r	-.173^**^	-.150^**^	-.298^**^	.378^**^	.355^**^	-.281^**^	.416^**^	.014	-.074	.083	.152^**^	.200^**^	.346^**^	.675^**^
p	**.001**	**.005**	**.000**	**.000**	**.000**	**.000**	**.000**	.791	.167	.121	**.004**	**.000**	**.000**	**.000**

(n=350).

Bold values in the table indicate that these indicators are statistically significant.

Common criteria are * for a significant level of less than 0.05 and ** for a significant level of less than 0.01.

### 3.3 Univariate analysis of GD group

Univariate analyses of the factors to predict elevated BTMs were given in **Table 3**. Regarding the elevated OST, the results showed that GD cases with higher levels of thyroxine, TRAb, Ca and ALP, lower levels of TSH, higher RAIU, larger W and shorter duration of disease would lead to abnormally elevated OST (P<0.05). In case of P1NP, the results showed that GD patients with higher levels of FT3, FT4, TRAb, Ca and ALP, lower levels of TSH and shorter duration of disease would lead to an abnormally elevated P1NP (P<0.05). Moreover, the results indicated that GD cases with elder age, higher levels of thyroxine, TRAb, Ca, ALP and 25(OH)-VD, lower levels of TSH, larger W and shorter duration of disease would lead to an abnormally elevated CTX-1 (P<0.05). Besides, the results above suggested that male patients with GD were more likely to have elevated P1NP and CTX-1 (P<0.05).

**Table 3:** Univariate analysis of BTMs.

Baseline factors	Univariate			
	Normal-OST	Elevated-OST	z	P
Gender(Male/Female)	16/56	86/192	-1.426	0.154
Age	33 (30, 37)	33 (27, 38)	-0.671	0.502
Duration of disease	60 (12, 120)	12 (3, 48)	-4.351	**<0.001**
FT3	11.05 (7.31, 19.74)	27.04 (14.95, 30.72)	-6.944	**<0.001**
FT4	21.49 (15.29, 30.42)	34.85 (25.67, 44.96)	-6.378	**<0.001**
TSH	0.004 (0.004, 0.006)	0.004 (0.004, 0.004)	-0.386	**<0.001**
TRAb	14.55 (6.97, 23.51)	26.51 (17.48, 40.00)	-0.6216	**<0.001**
TgAb	20.00 (20.00, 94.20)	33.2 (20.0, 224.0)	-1.624	0.104
TPOAb	235.00 (39.10, 830.00)	351.00 (59.6, 1000.00)	-1.551	0.121
RAIU	0.66 (0.59, 0.71)	0.69 (0.62, 0.74)	-2.273	**0.023**
Thyroid weight	24.5 (17.2, 41.0)	30.85 (22.5, 45.8)	-2.206	**0.027**
25(OH)-VD	30.82 (24.48, 43.29)	31.55 (24.2, 46.24)	-0.763	0.446
Ca	2.32 (2.27, 2.41)	2.41 (2.35, 2.50)	-5.781	**<0.001**
ALP	69.00 (58.00, 81.00)	111.50 (83.75, 148.25)	-8.7	**<0.001**

Baseline factors	Univariate			
	Normal-P1NP	Elevated-P1NP	z	P
Gender(Male/Female)	15/73	88/174	-2.942	**0.003**
Age	33.00 (29.00, 38.75)	33.5 (28.0, 38.0)	-0.201	0.841
Duration of disease	48 (12, 117)	9.5 (3.0, 48.0)	-5.103	**<0.001**
FT3	13.410 (7.665, 23.350)	27.5600 (14.1825, 30.7200)	-5.945	**<0.001**
FT4	26.5500 (17.1325, 34.7025)	34.4750 (25.0950, 44.7075)	-4.858	**<0.001**
TSH	0.00400 (0.00400, 0.00775)	0.004 (0.004, 0.004)	-5.554	**<0.001**
TRAb	16.86 (8.5225, 27.8825)	26.2900 (17.3125, 39.7000)	-5.008	**<0.001**
TgAb	28.30 (20.00, 168.25)	30.6 (20.0, 205.0)	-0.428	0.669
TPOAb	243.000 (41.825, 1000.000)	350.50 (58.75, 1000.00)	-1.141	0.254
RAIU	0.685 (0.610, 0.720)	0.685 (0.610, 0.740)	-0.79	0.43
Thyroid weight	18.525 (25.800, 41.900)	30.9 (22.4, 45.8)	-1.941	0.052
25(OH)-VD	29.5150 (23.8925, 40.9225)	32.4450 (24.6325, 48.3825)	-1.836	0.066
Ca	2.34 (2.29, 2.41)	2.42 (2.35, 2.50)	-5.321	**<0.001**
ALP	66.00 (54.25, 77.75)	114.0 (89.5, 152.0)	-10.556	**<0.001**

Baseline factors	Univariate			
	Normal-CTX-1	Elevated-CTX-1	z	P
Gender(Male/Female)	30/107	73/140	-2.543	**0.011**
Age	34.00 (30.00, 39.25)	33.00 (27.00, 38.00)	-2.346	**0.019**
Duration of disease	48 (5, 96)	10 (3, 36)	-4.377	**<0.001**
FT3	15.635 (9.3075, 20.7630)	30.6250 (15.1975, 30.7200)	-5.741	**<0.001**
FT4	27.8150 (18.2625, 35.1475)	36.3850 (26.4275, 45.3825)	-5.336	**<0.001**
TSH	0.004 (0.004, 0.004)	0.004 (0.004, 0.004)	-4.04	**<0.001**
TRAb	18.4000 (10.9350, 26.5275)	28.135 (20.1825, 40.0000)	-6.125	**<0.001**
TgAb	30.5 (20.0, 237.0)	28.10 (20.00, 160.75)	-0.281	0.779
TPOAb	243.00 (59.45, 1000.00)	406.5 (52.425, 1000.000)	-0.893	0.372
RAIU	0.68 (0.61, 0.72)	0.69 (0.61, 0.75)	-1.13	0.258
Thyroid weight	26.10 (19.30, 43.65)	31.05 (22.6075, 45.8000)	-1.979	**0.048**
25(OH)-VD	28.510 (23.815, 40.480)	33.695 (24.6775, 49.425)	-2.271	**0.023**
Ca	2.36 (2.29, 2.45)	2.42 (2.36, 2.50)	-5.131	**<0.001**
ALP	74.0 (60.0, 96.5)	121.00 (93.25, 163.75)	-10.284	**<0.001**

Bold values in the table indicate that these indicators are statistically significant.

### 3.4 ROC curve analysis and predictive value of selected indicators for elevated BTMs

Based on the results obtained from the above univariate analyses, we further assessed date with statistical significant by ROC curve (**Table 4**). It was shown that duration of disease, RAIO, W and the levels of FT3, FT4, TSH, TRAb, Ca and ALP had good performances in predicting elevated OST. In the prediction of elevated P1NP, duration of disease and the levels of FT3, FT4, TSH, TRAb, Ca and ALP had good performances. In the prediction of elevated CTX-1, age, duration of disease, the levels of FT3, FT4, TSH and TRAb, W and the levels of 25(OH)-VD, Ca and ALP had good performances.

**Table 4:** ROC curve analysis counting indicators of univariate analysis to predict elevated BTMs.

Indicators	AUC	Standard Error	P	95%		Cut-off	Sensitivity	Specificity
				Confidence Interval				
				Lower Bound	Upper Bound			
Duration of disease	0.667	0.04	<0.001	0.588	0.745	<21	0.69	0.595
FT3	0.759	0.032	<0.001	0.696	0.823	>15.765	0.737	0.732
FT4	0.746	0.033	<0.001	0.681	0.811	>31.37	0.612	0.789
TSH	0.588	0.041	0.022	0.508	0.668	<0.0045	0.268	0.9
TRAb	0.738	0.034	<0.001	0.671	0.806	>20.37	0.694	0.69
RAIU	0.584	0.036	0.03	0.512	0.655	>0.715	0.363	0.789
Thyroid weight	0.585	0.042	0.027	0.502	0.668	>22.35	0.763	0.479
Ca	0.723	0.033	<0.001	0.658	0.788	>2.345	0.77	0.604
ALP	0.834	0.023	<0.001	0.789	0.88	>87.5	0.73	0.831
Duration of disease	0.681	0.035	<0.001	0.613	0.75	<21	0.716	0.622
FT3	0.706	0.032	<0.001	0.643	0.77	>15.765	0.732	0.625
FT4	0.674	0.034	<0.001	0.608	0.74	>30.46	0.628	0.67
TSH	0.617	0.037	0.001	0.544	0.69	<0.0045	0.307	0.934
TRAb	0.678	0.034	<0.001	0.611	0.745	>20.37	0.697	0.625
Ca	0.69	0.032	<0.001	0.627	0.754	>2.345	0.77	0.534
ALP	0.876	0.02	<0.001	0.836	0.916	>85.5	0.793	0.864
Age	0.574	0.031	0.019	0.514	0.634	<28.5	0.819	0.33
Duration of disease	0.638	0.031	<0.001	0.577	0.7	<42	0.507	0.774
FT3	0.673	0.029	<0.001	0.615	0.731	>26.515	0.561	0.75
FT4	0.666	0.03	<0.001	0.607	0.725	>33.09	0.585	0.713
TSH	0.426	0.032	0.02	0.363	0.489	<0.0045	0.225	0.925
TRAb	0.691	0.029	<0.001	0.634	0.748	>20.79	0.745	0.596
Thyroid weight	0.564	0.032	0.043	0.501	0.628	>25.85	0.67	0.5
25(OH)-VD	0.57	0.031	0.027	0.51	0.631	>32.515	0.542	0.518
Ca	0.66	0.03	<0.001	0.602	0.718	>2.355	0.759	0.485
ALP	0.826	0.022	<0.001	0.783	0.867	>88.5	0.807	0.684

Bold values in the table indicate that these indicators are statistically significant.

### 3.5 Multivariate logistic regression analysis

In GD group, multivariate logistic regression (**Table 5**) revealed that FT3 (OR:0.22, 95%CI: 0.075-0.643), W (OR:0.357, 95%CI: 0.160-0.797), Ca (OR:0.336, 95%CI: 0.156-0.721) and ALP (OR:0.121, 95%CI: 0.055-0.266) were independent factors in predicting the elevation of OST. And multivariate logistic regression revealed that duration of disease (OR:2.415, 95%CI: 1.206-4.838), FT3 (OR:0.246, 95%CI: 0.087-0.691), TSH (OR:2.983, 95%CI: 1.199-7.418) and ALP (OR:0.062, 95%CI: 0.029-0.133) were independent factors in predicting the elevation of P1NP. Besides, age (OR:3.106, 95%CI: 1.583-6.092), duration of disease (OR:2.387, 95%CI: 1.292-4.411), TRAb (OR:0.525, 95%CI: 0.292-0.946) and ALP (OR:0.175, 95%CI: 0.098-0.311) were independent factors in predicting the elevation of CTX-1.

**Table 5:** Logistic regression analyses of factors to predict elevated BTMs.

Normal-OST/ Elevated-OST	Multivariate			
	P	OR	95%CI	
Duration of disease (<21/≥21)	0.068	2.046	0.947	4.417
**FT3** (<15.765/≥15.765)	**0.006**	0.22	0.075	0.643
FT4 (<31.37/≥31.37)	0.85	1.113	0.367	3.372
TSH (<0.0045/≥0.0045)	0.679	0.822	0.323	2.086
TRAb (<20.37/≥20.37)	0.204	0.616	0.292	1.3
Radioactive iodine uptake rate (<0.715/≥0.715)	0.125	0.528	0.234	1.194
**Thyroid weight** (<22.35/≥22.35)	**0.012**	0.357	0.16	0.797
**Ca** (<2.345/≥2.345)	**0.005**	0.336	0.156	0.721
**ALP** (<87.5/≥87.5)	**<0.001**	0.121	0.055	0.266

Normal-P1NP/ Elevated-P1NP	Multivariate			
	P	OR	95%CI	
Gender(Male/Female)	0.087	2.06	0.901	4.709
**Duration of disease** (<21/≥21)	**0.013**	2.415	1.206	4.838
**FT3** (<15.765/≥15.765)	**0.008**	0.246	0.087	0.691
FT4 (<30.46/≥30.46)	0.517	1.406	0.502	3.942
**TSH** (<0.0045/≥0.0045)	**0.019**	2.983	1.199	7.418
TRAb (<20.37/≥20.37)	0.63	0.842	0.419	1.692
Ca (<2.345/≥2.345)	0.197	0.616	0.295	1.286
**ALP** (<85.5/≥85.5)	**<0.001**	0.062	0.029	0.133

Normal-CTX-1/ Elevated-CTX-1	Multivariate			
	P	OR	95%CI	
Gender(Male/Female)	0.167	1.584	0.824	3.043
**Age** (<28.5/≥28.5)	**0.001**	3.106	1.583	6.092
**Duration of disease** (<42/≥42)	**0.005**	2.387	1.292	4.411
FT3 (<26.515/≥26.515)	0.219	0.598	0.264	1.356
FT4 (<33.09/≥33.09)	0.251	0.632	0.89	1.383
TSH (<0.0045/≥0.0045)	0.135	1.908	0.818	4.454
**TRAb** (<20.79/≥20.79)	**0.032**	0.525	0.292	0.946
Thyroid weight (<25.85/≥25.85)	0.23	0.675	0.356	1.281
25(OH)-VD (<32.515/≥32.515)	0.421	0.789	0.444	1.404
Ca (<2.355/≥2.355)	0.354	0.741	0.394	1.395
**ALP**(<88.5/≥88.5)	**<0.001**	0.175	0.098	0.311

Bold values in the table indicate that these indicators are statistically significant.

### 3.6 Plotting of alignment diagram and decision curve analysis

**Figure 3:**
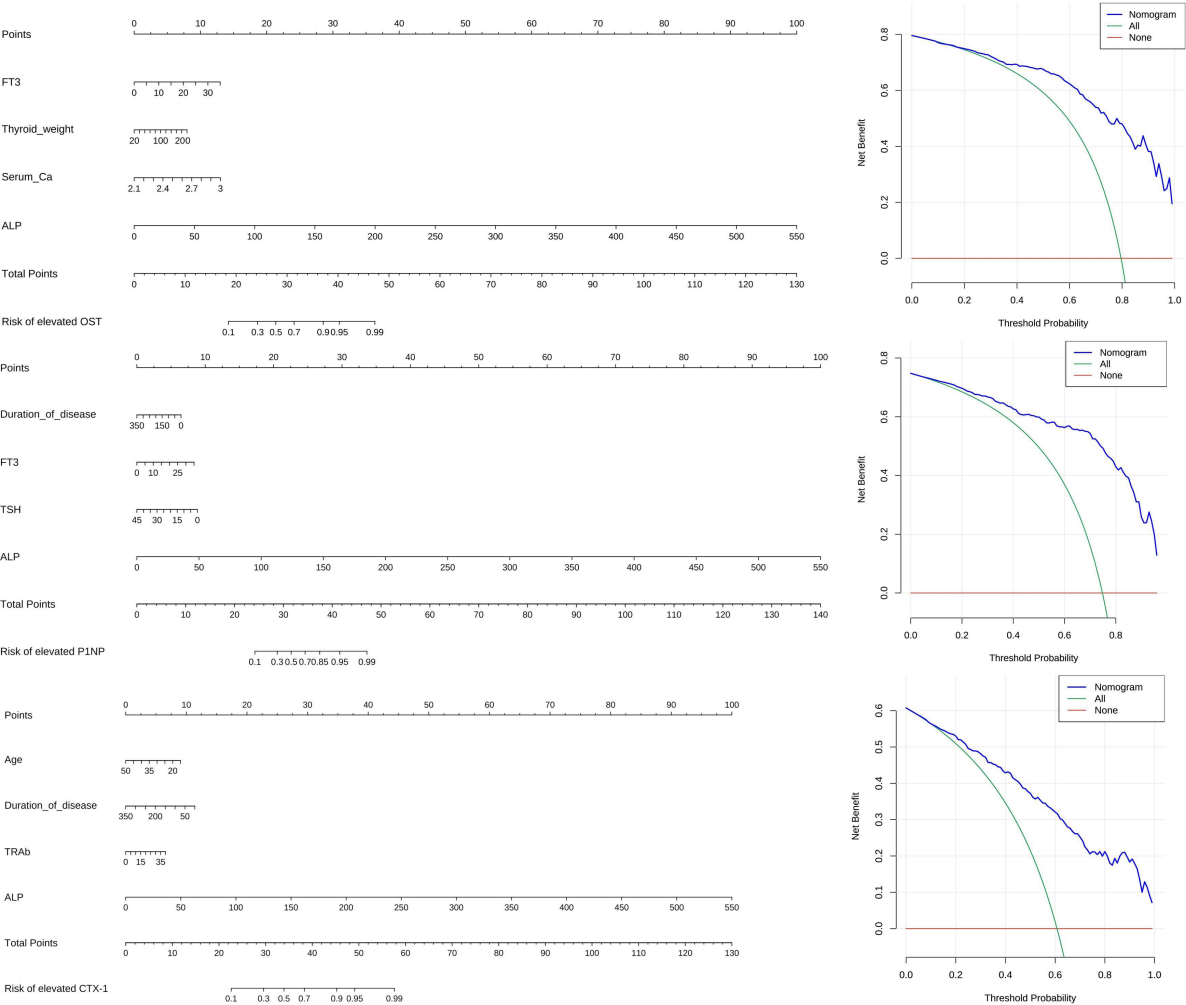
Alignment diagram and DCA.

We integrated the independent risk factors obtained after the above multi-factor logistic regression analysis to construct the Alignment Diagram (**Figure 3**). From the DCA of the Alignment Diagram model, the threshold probability range, in which the net benefit of the decision curve of this model was higher than the two invalid lines, is 14% to 100% for OST, 5% to 97% for P1NP and 4% to 100% for CTX-1 respectively.

### 3.7 Change of BTMs during treatment

**Figure 4:**
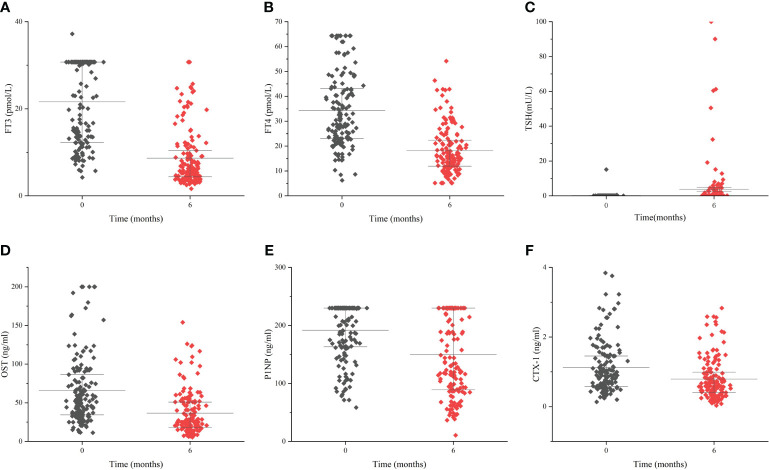
The change of thyroid hormones and bone turnover markers during the follow-up period Levels of FT3 **(A)**, FT4 **(B)**, TSH **(C)**, OST **(D)**, P1NP **(E)** and CTX-1 **(F)** during the follow-up period of 6 months. Data are presented as median and interquartile range.

During the treatment, the levels of FT3, FT4 declined, meanwhile the level of TSH increased. The values of these indicators became normalized (**Table 6**, **Figures 4A–C**). Regarding markers of bone metabolism, there was a marked decrease in OST from 52.4 (34.1, 88.3) at baseline to 26.7(18.2, 50.9) at follow-up visit, along with P1NP from 230.0 (162.4, 230.0) to 135.6 (88.8, 230.0) and OST from 0.904 (0.570, 1.453) to 0.603 (0.399, 0.994) (**Table 6**, **Figures 4D–F**).

**Table 6:** Thyroid hormone and BTMs (median and range) at initial diagnosis and follow-up for 6 months in 149 patients with GD.

	Baseline	follow-up for 6m	Z	P-Value
FT3 (pmol/L)	22.95(12.25, 30.72)	6.17(4.35, 10.67)	-11.496	0.000
FT4 (pmol/L)	30.42(23.02, 43.42)	15.22(11.88, 22.70)	-9.989	0.000
TSH (uIU/mL)	0.004(0.004, 0.004)	0.004(0.004, 1.130)	-7.734	0.000
OST (ng/ml)	52.4(34.1, 88.3)	26.7(18.2, 50.9)	-7.359	0.000
P1NP (ng/ml)	230.0(162.4, 230.0)	135.6(88.8, 230.0)	-5.325	0.000
CTX-1 (ng/ml)	0.904(0.570, 1.453)	0.603(0.399, 0.994)	-4.678	0.000

### 3.8 Predictive value of degree of decrease in thyroxine for recovery of BTMs

**Figure 5:**
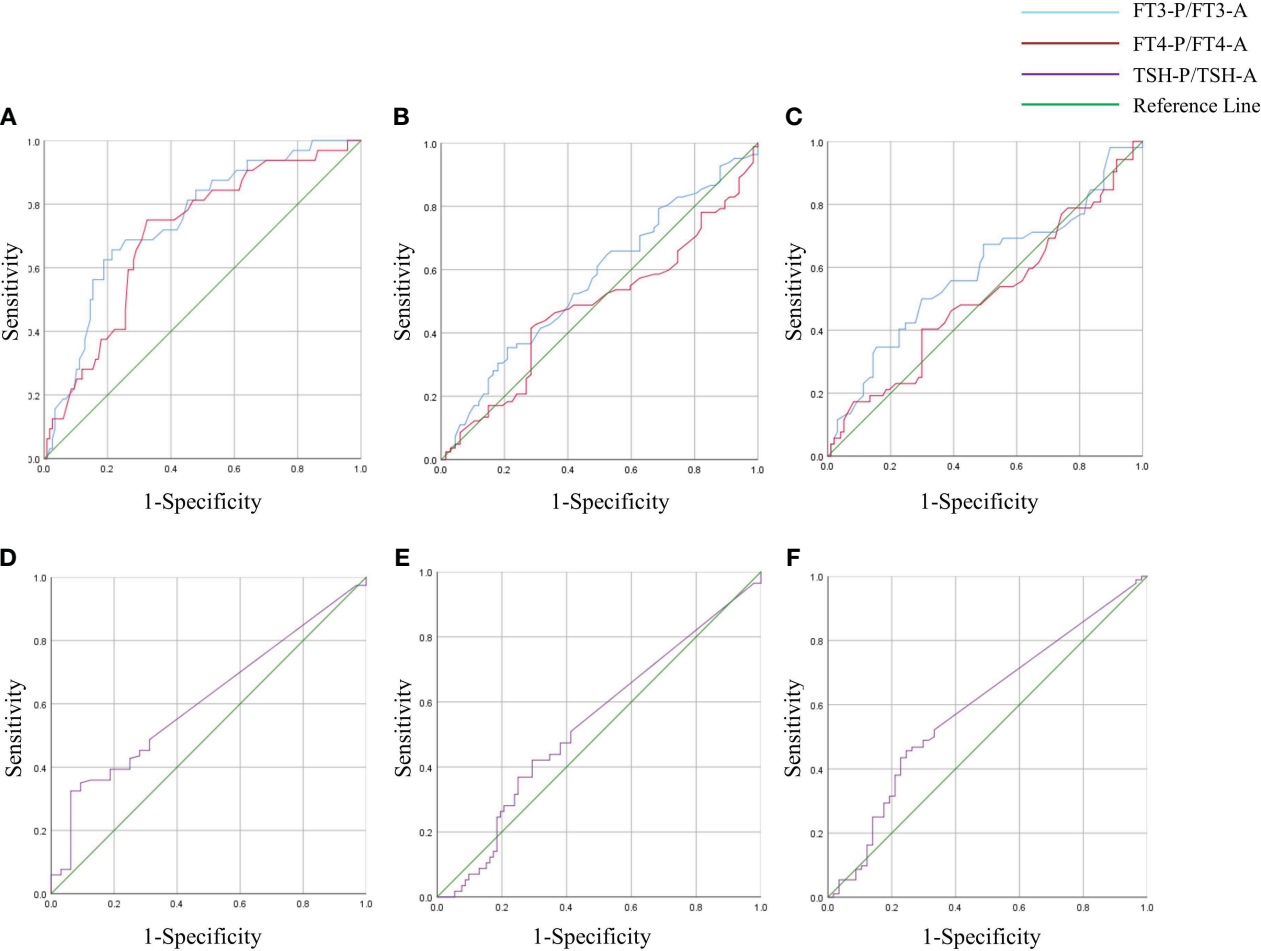
ROC curve of FT3-P/ FT3-A, FT4-P/ FT4-A and TSH-P/TSH-A in GD group. Degree of decrease in thyroxine (FT3-P/ FT3-A, FT4-P/ FT4-A and TSH-P/TSH-A) were used as test variables to plot receiver operating characteristic(ROC) curve with whether BTMs(including OST, P1NP and CTX-1) improved as status variables. The state variables in panels **(A)** and **(D)** were whether OST improved. The state variables in panels **(B)** and **(E)** were whether P1NP improved. The state variables in panels **(C)** and **(F)** were whether CTX-1 improved.

The degree of decrease in thyroxine (FT3-P/FT3-A、FT4-P/FT4-A and TSH-P/TSH-A) were used as test variables to plot ROC curve with whether BTMs(including OST, P1NP and CTX-1) improved as status variables. The results showed that the AUC for FT3-P/FT3-A and FT4-P/FT4-A was 0.748 and 0.705 to predict the recovery of OST, meanwhile the cut-off value was 0.51 and 0.595 respectively. (P < 0.05). The degree of decrease in TSH had no predictive value for recovery of OST (P > 0.05).The degree of decrease in thyroxine had no predictive value for recovery of P1NP and CTX-1 (P > 0.05) (**Figure 5**, **Table 7**).

**Table 7:** ROC curves for the degree of decrease in thyroxine to predict recovery of BTMs.

Indicators	AUC	Standard Error	P	95%		Cut-off	Sensitivity	Specificity
				Confidence Interval				
				Lower Bound	Upper Bound			
FT3-P/ FT3-A	0.748	0.047	**<0.001**	0.656	0.84	<0.51	0.656	0.786
FT4-P/ FT4-A	0.705	0.05	**<0.001**	0.608	0.803	<0.595	0.75	0.675
TSH-P/TSH-A	0.61	0.052	0.057	0.508	0.712	–	–	–
FT3-P/ FT3-A	0.567	0.047	0.163	0.474	0.659	–	–	–
FT4-P/ FT4-A	0.481	0.048	0.684	0.387	0.574	–	–	–
TSH-P/TSH-A	0.534	0.049	0.489	0.438	0.629	–	–	–
FT3-P/ FT3-A	0.585	0.051	0.086	0.485	0.685	–	–	–
FT4-P/ FT4-A	0.507	0.051	0.8894	0.406	0.607	–	–	–
TSH-P/TSH-A	0.593	0.048	0.056	0.499	0.688	–	–	–

Bold values in the table indicate that these indicators are statistically significant.

## 4 Discussion

Bone homeostasis depended on the dynamic regulation of osteoblasts and osteoclasts, and it was rigorously regulated by a local feedback loop concerning various cytokines, growth factors and many other elements. Thyroid hormone is irreplaceable in this process. It has deep effects on skeletal development, linear growth and bone mass maintenance, which has been recognized for over one hundred years (5).

If the body produced too much thyroid hormone, the metabolism of bones may accelerate. This phenomenon could be observed even in patients with subclinical hyperthyroidism (7, 19). The metabolism of bones, even all bone cell types, was strongly controlled by the Wnt signaling pathway (20). It was found that thyroid hormone can regulate Wnt signaling in osteoblasts (6). And TSH, a hormone closely related to thyroid development and thyroid hormone secretion, stimulates the production of Wnt5a that not only causes an increase in osteoblasts but also produces OPG, which can further increase bone mass by inhibiting the resorption of osteoclast (21, 22). The accelerated metabolism of bones, both bone formation and bone resorption, may further lead to secondary osteoporosis, which could be mainly diagnosed by DXA measurement of BMD and greatly reduces the quality of life of patients (7, 9, 10, 12, 23). This theory was further confirmed by dynamic bone histomorphometry experiments conducted by Elena Tsourdi et al. in a study on the effects of hyperthyroidism on bone mass and bone turnover (6). Pia Nicolaisen et al. studied the effect of hyperthyroidism on bone microarchitecture and found that hyperthyroid women had lower vBMD and radial bone strength, as well as altered cortical microarchitecture, as compared to healthy controls. These abnormal changes improved significantly after one year of euthyroidism (24). This trend was similar to the results of our study. Because of timely diagnosis and treatment, severe osteoporosis caused by uncontrolled GD is a rarity in recent years (25).

Both BMD and BTMs are independent predictors of fracture risk. Actually, the use of BMD to determine the status of bone metabolism has a certain lag: substantial changes in BMD often occur six months after the metabolic abnormality. And the DXA test scores can hardly reflect the relationship between bone resorption and bone formation. In this way, the monitoring of serum bone metabolism indicators shows its advantages: it can not only show changes in bone quality and quantity in a timely manner, it can also provide evidence to trace back to the root. Especially in terms of treatment effect monitoring, BTMs should be prioritized (14).

In a cross-sectional analysis of 350 patients with GD, we found that patients with FT3 ≥ 15.765 pmol/L, W≥ 22.35 g, blood calcium ≥ 2.345 mmol/L, and ALP ≥ 87.5 U/L were more likely to have elevated OST; patients with disease duration <21 months, FT3 ≥15.765 pmol/L, TSH <0.0045 mU/L, and ALP ≥85.5 U/L were more likely to have elevated P1NP; patients aged <28.5 years with disease duration <42 months, TRAb ≥20.79 IU/L, and ALP ≥88.5 U/L were more likely to have elevated CTX-1. Furthermore, the Alignment Diagram in this study integrated variables with good predictive performance, and represented the quantitative relationship between variables with multiple mutually parallel line segments in planar coordinates, and the probability of occurrence of abnormally elevated BTMs was obtained by using the corresponding points on Total points as the vertical line of the BTMs elevation risk scale axis. This approach quantified the likelihood and allowed clinicians, and even the patients themselves, to calculate the probability of abnormally active bone metabolism based on the above model to determine whether further tests related to bone metabolism were needed. The results of DCA also indicated that, within a reasonable threshold probability range, the model predicted abnormally elevated BTMs in GD patients with high net benefit and good clinical efficacy.

After the above analysis we found that FT_3_ and ALP were the best predictors of abnormally elevated BTMs. FT_3_ is the most reliable indicator for clinicians to determine the severity of GD. It also directly indicates that the degree of hyperthyroidism osteoporosis and GD condition are closely related. Alkaline phosphatase, an enzyme mainly produced by the liver and bones, is also often considered as one of the indicators of bone formation (26). It acts in bone and muscle progenitor cells mainly by affecting mitochondrial respiration and ATP production (27). In clinical practice, some patients have abnormally high-ALP with transaminases in the normal range and no significant improvement with liver-protective therapy. After this study, it is reasonable to assume that such patients, especially those with FT3, TRAb, and ALP all greater than cutoff values should be further examined for bone metabolic markers as well as DXA, which may lead to unexpected gains.

Antithyroid drugs and ^131^I therapy are the two most commonly used treatments in clinical practice today (2). Our study showed that abnormally elevated bone metabolism markers normalize over the course of treatment as thyroid hormone decreases, which was similar to the findings of the Meta-analysis conducted by Peter Vestergaard et al. (28). We further conducted a cohort study, which is the innovation of this study, and found that GD patients with a 51% decrease in FT3 and 59.5% decrease in FT4 after treatment had a greater likelihood of recovering their bone formation index (OST). However, reduced thyroxine had no predictive value for bone resorption markers. We consider that it may be due to the stronger modulation of osteoblast activity by thyroxine, whereas the regulation of osteoclasts is achieved indirectly by increasing the expression of RANKL in osteoblasts and the activity of other cytokines involved in osteoclastogenesis, including interleukin 6 (IL-6), IL-8 and prostaglandin E2 (PGE2) (5, 20, 29). So during the decline of serum thyroxine levels, it is the osteoblasts that first receive the signal and respond, and thus bone formation markers recover earlier than bone resorption markers.

Nowadays, numerous studies have shown that P1NP is closely associated with various bone metabolic diseases, tumor bone metastasis and multiple myeloma (30–32). And its detection is not affected by interfering factors such as food, circadian rhythms and hormones (33). Therefore, PINP is a high-quality new clinical diagnostic marker for bone metabolism-related diseases and has been recommended by the Bone Marker Standards Working Group (14). However, the calculations in this study showed that the decrease in thyroxine levels had no predictive value for the recovery of P1NP. We consider that it may be limited by the assay method: serum P1NP levels of some patients were detected at values outside the detection range (>230ng/ml) both before and after treatment, so that the degree of change could not be judged. It is also possible that the follow-up period was short and P1NP has not yet shown a significant trend. The IOF/IFCC suggests the use of a bone formation marker (s-PINP) and a bone resorption marker (s-CTX) as reference markers, the implementation of which still requires the establishment of an international standard in the future.

## 5 Conclusion

Bone formation indexes(OST and P1NP) and bone resorption indexes(CTX-1) were abnormally elevated in GD patients, and these bone metabolism-related indexes were significantly correlated with serum levels of FT3, FT4, TRAb, Ca, and ALP. For abnormal elevation of BTMs in GD patients, FT3 and ALP showed strong predictive value. GD patients with 51% decrease in FT3 and 59.5% decrease in FT4 after treatment may be able to recover their bone formation indexes (OST) more quickly. This has some predictive value and research prospects (34).

## 6 Limitation

The limitation of this study is the relatively small sample size, especially for cohort analysis. Graves’ Disease affects also to a significant extent older women, whose bone turnover levels were negatively influenced by postmenopausal hormone levels. Sex hormone levels, body mass, physical activity and occurrence of other autoimmune diseases (e.g. celiac disease, Crohn’s disease, ulcerative colitis, or autoimmune gastritis, influencing resorption) may affect bone metabolic levels in some ways. Consequently, these groups were not included in the study in order to control for variables. The follow-up period in this study was 6 months, which could be further increased in duration and number of follow-ups. And in the cohort analysis, we were only able to monitor the patients’ thyroid function levels due to the limitations of the conditions during the follow-up. Besides, in further studies, we plan to include measurement of bone mineral density in the follow-up, which will enhance this study.

## Data availability statement

The data that supports the findings of this study are available upon reasonable request from the corresponding author.

## Ethics statement

All procedures performed in studies involving human participants were in accordance with the ethical standards of the institutional and/or national research committee and with the 1964 Helsinki declaration and its later amendments or comparable ethical standards. Informed consent was obtained from all participants included in this study.

## Author contributions

MS: Data curation, Formal analysis, Writing – original draft. JC: Conceptualization, Writing – review & editing. WZ: Project administration, Writing – review & editing. QJ: Project administration, Writing – review & editing. JT: Project administration, Writing – review & editing. YH: Project administration, Writing – review & editing. RZ: Project administration, Writing – review & editing. JM: Data curation, Writing – review & editing. WL: Data curation, Writing – review & editing. TS: Data curation, Writing – review & editing. JR: Data curation, Writing – review & editing. LD: Methodology, Writing – review & editing. LL: Methodology, Writing – review & editing. ZM: Conceptualization, Writing – review & editing.
